# The HLA-B*57:01 allele corresponds to a very large *MHC* haploblock likely explaining its massive effect for HIV-1 elite control

**DOI:** 10.3389/fimmu.2023.1305856

**Published:** 2023-12-11

**Authors:** Myriam Rahmouni, Lorenzo De Marco, Jean-Louis Spadoni, Maxime Tison, Raissa Medina-Santos, Taoufik Labib, Josselin Noirel, Ryad Tamouza, Sophie Limou, Olivier Delaneau, Jacques Fellay, Armand Bensussan, Sigrid Le Clerc, Paul J. McLaren, Jean-François Zagury

**Affiliations:** ^1^ Laboratoire Génomique, Bioinformatique, et Chimie Moléculaire, EA7528, Conservatoire National des Arts et Métiers, HESAM Université, Paris, France; ^2^ Université Paris Est Créteil, INSERM U955, IMRB, Laboratoire Neuro-Psychiatrie translationnelle, Créteil, France; ^3^ Nantes Université, Ecole Centrale Nantes, INSERM, Center for Research in Transplantation and Translational Immunology (CR2TI), Nantes, France; ^4^ Department of Computational Biology, University of Lausanne, Lausanne, Switzerland; ^5^ Global Health Institute, School of Life Sciences, École Polytechnique Fédérale de Lausanne, Lausanne, Switzerland; ^6^ Unité INSERM U976, Hôpital Saint-Louis, Paris, France; ^7^ Sexually Transmitted and Blood-Borne Infections Division at JC Wilt Infectious Diseases Research Centre, National Microbiology Laboratory Branch, Public Health Agency of Canada, Winnipeg, MB, Canada; ^8^ Department of Medical Microbiology and Infectious Diseases, University of Manitoba, Winnipeg, MB, Canada

**Keywords:** elite controllers, HIV-1, AIDS, genetics, GWAS, viral load, MHC, haplotype

## Abstract

**Introduction:**

We have reanalyzed the genomic data of the International Collaboration for the Genomics of HIV (ICGH), centering on HIV-1 Elite Controllers.

**Methods:**

We performed a genome-wide Association Study comparing 543 HIV Elite Controllers with 3,272 uninfected controls of European descent. Using the latest database for imputation, we analyzed 35,552 Single Nucleotide Polymorphisms (SNPs) within the Major Histocompatibility Complex (*MHC*) region.

**Results:**

Our analysis identified 2,626 SNPs significantly associated (p<5. 10-8) with elite control of HIV-1 infection, including well-established *MHC* signals such as the rs2395029-G allele which tags HLA-B*57:01. A thorough investigation of SNPs in linkage disequilibrium with rs2395029 revealed an extensive haploblock spanning 1.9 megabases in the *MHC* region tagging HLA-B*57:01, comprising 379 SNP alleles impacting 72 genes. This haploblock contains damaging variations in proteins like NOTCH4 and DXO and is also associated with a strong differential pattern of expression of multiple *MHC* genes such as *HLA-B, MICB*, and *ZBTB12*. The study was expanded to include two cohorts of seropositive African-American individuals, where a haploblock tagging the HLA-B*57:03 allele was similarly associated with control of viral load. The mRNA expression profile of this haploblock in African Americans closely mirrored that in the European cohort.

**Discussion:**

These findings suggest that additional molecular mechanisms beyond the conventional antigen-presenting role of class I HLA molecules may contribute to the observed influence of HLA-B*57:01/B*57:03 alleles on HIV-1 elite control. Overall, this study has uncovered a large haploblock associated with HLA-B*57 alleles, providing novel insights into their massive effect on HIV-1 elite control.

## Introduction

Despite the availability of effective antiretroviral drugs, HIV continues to be an important public health concern, with a large number of new infections and deaths occurring each year, particularly in low-income countries (1) In the early 2000s, a subset of HIV-positive individuals who maintained consistently low viral loads for several years without receiving any treatment were identified and referred to as Elite Controllers (ECs). These individuals represent approximately 0.2 to 0.5% of the HIV-positive population (2), including in African cohorts (3), a finding that has been confirmed in the GRIV cohort comprising Long-Term Non-Progressors and ECs (see methods). Genetic association studies initially revealed a strong association between EC status and the presence of the HLA-B*57:01 allele (4, 5). Subsequently, large-scale genome-wide association studies (GWAS) confirmed that genetic variants within the Major Histocompatibility Complex (MHC) region exerted the most significant genetic influence on viral load control and disease progression (6, 7). In 2010, a GWAS focusing on ECs identified four independent top signals, all localized within the HLA region (8). In 2013, the ICGH consortium, a global collaborative effort comprising multiple AIDS GWAS studies published a first large genome-wide association study comparing 6,300 HIV-1 infected individuals and 7,200 uninfected controls and found no difference except in the well-known CCR5 region (9). In 2015, the same ICGH consortium studied genetic associations with the viral load at setpoint among the 6,300 HIV-1 infected individuals and found signals mostly in the MHC and to a lesser extent in the CCR5 region, reaffirming the prominent impact of the HLA region on viral load and disease progression (10). However, since then, no new findings regarding this region have emerged. Given that EC subjects possess natural control over viral infection, they represent a valuable population for investigating the molecular mechanisms of protection. So far, the explanations for the biological impact of the MHC region in HIV-1 control have been limited to the role of classical HLA class I alleles, HLA-A, HLA-B, and HLA-C (10–13). To delve deeper into the understanding of the MHC region’s biological impact on HIV-1 control, we reanalyzed the genetic data of 543 ECs (with viral loads<1,000 copies/ml) and 3,272 uninfected controls from the ICGH consortium (references IHAC1/2), with a specific focus on the MHC region. Considering that ECs constitute 0.2-0.5% of the seropositive population (2), this group encompasses an extreme phenotype representing over 100,000 seropositive individuals. Leveraging the latest bioinformatics databases, we imputed 35,552 Single Nucleotide Polymorphisms (SNPs) within the MHC region, and identified 2,626 significant signals surpassing the genome-wide significance threshold (5.10-8). The MHC region spans approximately 5 million base pairs (genomic coordinates 28,477,797 to 33,448,354) on chromosome 6, according to the latest GENCODE gene annotation for the GRCh38 reference genome (14). Within this region, a total of 373 protein-coding genes, 18 pseudogenes, and 12 non-coding RNA genes were identified by the GENCODE annotation.

To translate these 2,626 SNP associations into function, we then conducted a comprehensive analysis of these positive signals with the goal of addressing the following key biological questions, namely, **a.** the reason for the massive genetic impact of the MHC region in AIDS, **b.** the factors explaining why only a fraction of HLA-B*57:01 individuals are Elite Controllers, and **c.** the understanding of the molecular mechanisms of protection in Elite Controllers.

## Results

### Huge number of MHC SNPS associating to elite control in AIDS

**Figure 1:**
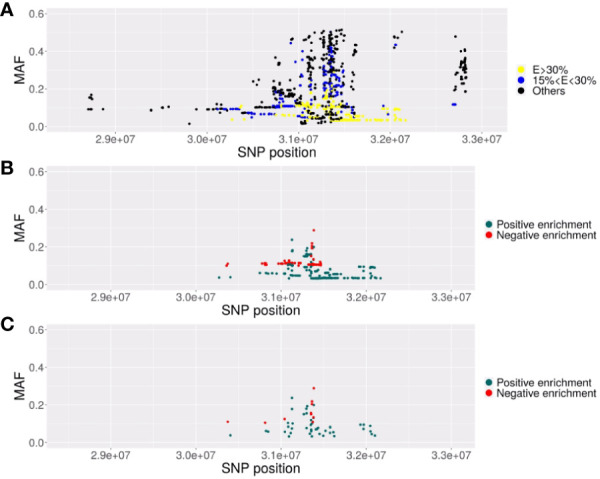
Description of the SNPs associated with EC in the *MHC* region. Representation of the SNPs with their localization in chromosome 6 GRCh38) (x-axis) and their MAF (y-axis). **(A)** Representation of the 2626 SNPs significantly associated (p<5. 10^-8^) with elite control in the EC vs CTR GWAS. In yellow, the SNPs with minor alleles highly enriched (increase>30%) in EC2 subjects (VL<100 copies/ml) compared to EC3 subjects (VL comprised between 100 and 1000 copies/ml) or highly decreased (decrease of the MAF higher than 30% in EC2 compared to EC3). In blue, SNPs with a moderate enrichment or decrease (between 15 to 30%). **(B)** Representation of the 379 SNPs significant in the GWAS and highly enriched (E>30%) (in blue) or highly decreased (in red). **(C)** Representation of the 54 SNPs remaining after clumping at r2>=0.8, representative of the previous 379 significant SNPs composing the haploblock.

The 543 ECs (VL< 1,000 copies/ml) and 3,272 controls (CTR) from ICGH originated from 2 series of case-control individuals genotyped on two Illumina chips (**Table 1A**). We could impute 35,552 SNPs with an info score test > 0.75 for all the case and control individuals in the MHC region (see Methods). We compared a total of 543 ECs with 3,272 control individuals using a meta-analysis on the 2 case-control comparisons (see Methods) and identified 2626 significant SNPs (p<5.10-8) out of the 35,552 SNPs tested. Among these SNPs, the top four signals previously reported by Pereyra et al. (8) were confirmed (**Table 1B**) and also replicated in the independent GRIV study involving 50 ECs (15). Interestingly, by stratifying our EC sample into those with viral loads below 100 copies/ml (referred to as EC2, N=217) and those with viral loads between 100 and 1,000 copies/ml (referred to as EC3, N=326), we noticed a strong enrichment (>30%) of the protective minor allele for rs2395029 corresponding to HLA-B*57:01 and rs4418214 corresponding to MICA (**Table 1B**). To prioritize the most relevant SNPs among the 2,626 SNPs found significant in the MHC region, we thus decided to focus on those exhibiting a similar enrichment, i.e. those with a frequency difference >30% between EC2 and EC3. Out of the 2,626 SNPs, 379 showed an enrichment >30% between EC2 (VL<100 copies/ml) and EC3 (VL between 100 and 1,000 copies/ml) (**Figures 1A, B**). To enhance the precision of this analysis, we applied clumping to this group of 379 SNPs using an r2 threshold of 0.8, resulting in 54 representative SNPs ranging from position 30,374,976 to position 32,103,233 (**Figure 1C**). Among these representative SNPs, 44 showed a protective effect (higher minor allele frequency in EC2 compared to EC3 and controls) and 10 SNPs showed a negative effect (lower minor allele frequency in EC2 compared to EC3 and controls). The distribution of these 54 SNPs was consistent across the two ICGH case groups, as well as in the two control groups.

**Table 1:** Description of the cohorts and main signals of Pereyra et al. (8).

A.								
Groups	origins	Platform	N(EC)	HLA-B*57:01	EC2	EC2_HLA-B*57:01	EC3	EC3_HLA-B*57:01
ill1	North America and non-Dutch	Illumina 550 Illumina 650 Illumina 1M	418	119 (28%)	184	66 (36%)	234	53 (23%)
ill2	Non-French European		125	27 (22%)	33	9 (27%)	92	18 (20%)
**TOTAL**			543	146 (27%)	217	75 (35%)	326	71 (22%)
Groups	Origins	Platform	N (CTR)			HLA-B*57:01		
CTR1	North America and non-Dutch	Illumina 550 Illumina 650 Illumina 1M	2759			185 (6,7%)		
CTR2	Non-French European		513			25 (4,8%)		
**TOTAL**			3272			210 (6,4%)		
Groups	Origins	Platform	N (HIV+)			HLA-B*57:03	EC2	EC3
cs1	African American	Illumina 650	380			65 (17%)	43	67
cs2	African American	Illumina 1M	379			66 (17%)	45	43
**TOTAL**			759			131 (17%)	88	110
B.								
SNP	Gene	MAF CTR	MAF EC	MAF EC3	MAF EC2	Enrichment	P; OR EC vs CTR	P; OR Pereyra et al.
rs9264942	*LINC02571*	38.5%	58.1%	55.7%	61.7%	10.9%	2.91E-32; 2.2	2.81E-35; 2.9
rs4418214	*MICA*	7.2%	24.4%	21.2%	29%	**37.1%**	3.89E-63; 4.2	1.40E-34; 4.4
rs2395029	*HCP5*	3.3%	13.9%	11.5%	17.5%	**52.3%**	1.24E-44; 4.8	9.70E-26; 5.3
rs3131018	*PSOR1C3*	36.3%	22.7%	22.4%	22.8%	0%	9.84E-18; 1.9	4.210-16; 2.1

**A**. Description of the populations used in the study and the genotyping platforms, including the number of super-elite controlers with a viral load below 100 copies/ml (EC2) or regular elite controlers with a viral load comprised between 100 and 1000 copies/ml (EC3). This table also confirms the enrichment of subjects carrying the HLA-B*57:01 allele among the various EC populations. There is an enrichment of HLA-B*57:03 in the African-American Cs1 and Cs2 seropositive cohorts caused by the adjunction of additional Ecs. **B**. The 4 main independent SNP identified by Pereyra et al. 8 in MHC are also found in our EC-CTR case control study with similar p values. We have also specified the enrichment observed for these SNPs in the EC2 group compared to the EC3 group, one can see it is higher than 30% for SNPs rs4418214 and rs2395029.

### A large haploblock is in complete LD with the HLA-B*57:01 allele

**Figure 2:**
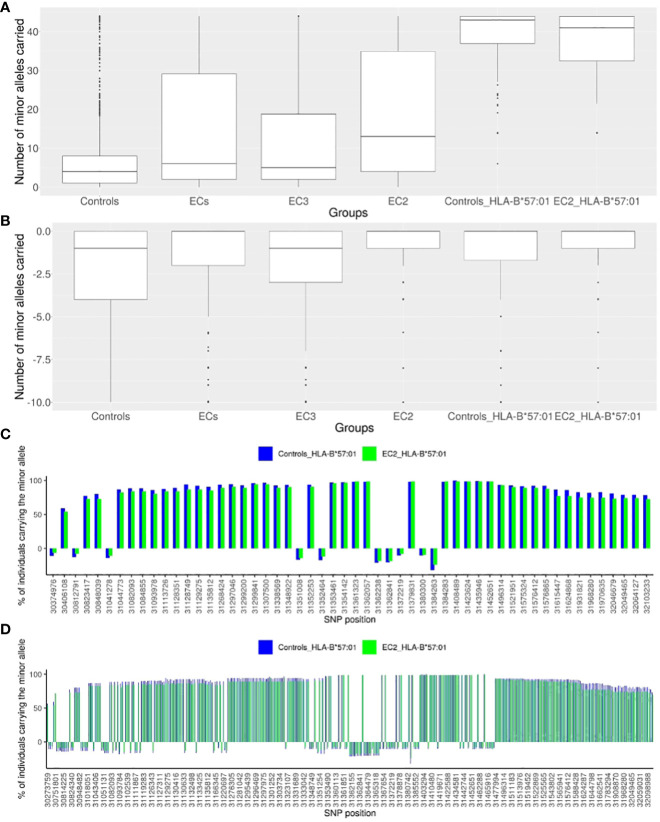
Description of the representative SNPs of the European haploblock. **(A)** Box-plot presenting the numbers of minor alleles of the 44 enriched representative SNPs carried for each individual in a given group: the CTR population (N=2443), the EC group (N=543), the EC2 group (N=217), the EC3 group (N=326), the CTR-HLA-B*57:01 group (N=110), the EC2-HLA-B*57:01 group (N= 75). **(B)** Box-plot presenting the numbers of minor alleles of the 10 decreased representative SNPs carried for each individual in a given group: the CTR population (N=2443), the EC group (N=543), the EC2 group (N=217), the EC3 group (N=326), the CTR-HLA-B*57:01 (CTR who are HLA-B*57:01) group (N=110), the EC2-HLA-B*57:01 (EC2 who are HLA-B*57:01) group (N= 75). **(C)** Histogram presenting the percentage of individuals carrying each minor allele of the 54 representative SNPs: for the CTR-HLA-B*57:01 group (N=110) in blue, and for the EC2-HLA-B*57:01 group (N=75) in green. The SNPs are represented by their localization (GRCh38) on the x-axis. For all SNPs, there is no significant difference observed between the CTR-HLA-B*57:01 and EC-HLA-B*57:01 groups. **(D)** Histogram presenting the percentage of individuals carrying each minor allele of the 379 SNPs of the haploblock: for the CTR-HLA-B*57:01 group (N=110) in blue, and for the EC2-HLA-B*57:01 group (N=75) in green. The SNPs are represented by their localization in chromosome 6 (GRCh38) on the x-axis.

For each individual, we then computed 2 scores as the number of carried “protective” alleles (from the 44 SNPs), or alternatively, the number of carried “risk” alleles (from the 10 negatively enriched SNPs). **Figures 2A, B** depict the box-plot representation of the scores obtained in the ECs (regrouped together as a single group) and in the CTR (regrouped together as a single group). As expected, the CTR individuals globally carry fewer protective alleles than the EC individuals, and also fewer than EC3 and much fewer than EC2 subjects.

To address the question of why only 1-2% of HLA-B*57:01 individuals are Elite Controllers (see Methods), we compared the 210 CTR-HLA-B*57:01 subjects with the 75 EC2-HLA-B*57:01 and 71 EC3-HLA-B*57:01 subjects with respect to each of the 54 variants (**Figure 2**). **Figure 2A** shows that the CTR-HLA-B*57:01 subjects carry most of the protective variants (median is 43 out of 44 possible variants), as many as the EC2-HLA-B*57:01 (or EC3-HLA-B*57:01 subjects, not shown). We thus decided to check whether the distribution of the 54 variants was similar between CTR-HLA-B*57:01 and EC2-HLA-B*57:01. **Figure 2C** illustrates the proportion of individuals carrying the protective (or negative) alleles for each SNP. Remarkably, there was no significant difference observed for any SNP between CTR-HLA-B*57:01 and EC2-HLA-B*57:01. Overall, **Figure 2** demonstrates the existence of a huge haploblock that encompasses almost all of the 44 positive SNPs and the major alleles of the 10 negative SNPs, which are carried concurrently by the majority of HLA-B*57:01 individuals, both in the CTR and EC2 groups. As shown in **Figure 2C**, the carriers of these variants were higher than 59% on the left side of the figure and higher than 78% on the right side, indicating a slight loss of linkage disequilibrium (LD) compared with the middle region (peak at 100%) corresponding to SNP rs112515516 (position 31,361,323), a representative of HLA-B*57:01.

Overall, the 379 enriched SNPs before the clumping form a haploblock ranging from position 30,273,759 to position 32,168,252 (from rs28894080 to rs1802036 SNPs). **Figure 2D** illustrates that the minor alleles of these SNPs within the haploblock are carried by at least 50% and often by 75% to 100% of HLA-B*57:01 carrying individuals. Overall, HLA-B*57:01 individuals carry a substantial proportion of the minor alleles of the 379 SNPs simultaneously, and there was no significant difference observed between CTR-HLA-B*57:01 and EC-HLA-B*57:01 individuals in terms of distribution of these alleles. In summary, we observe a remarkably large haploblock corresponding to the HLA-B*57:01 allele in Europeans: it spans 1,894,493 base pairs and involves 379 SNPs in LD (**Figure 2D**). Interestingly, there was no significant signal when comparing by GWAS the ECs carrying the HLA-B*57:01 allele (EC-HLA-B*57:01) with the uninfected controls carrying the HLA-B*57:01 allele (CTR-HLA-B*57:01).

Detailed information about these variants and those in high LD (r^2^>0.8) can be found in **Supplementary Table 1**. These variants correspond to SNPs within or close to genes therefore having the potential to impact 72 genes (**Supplementary Table 1A**), SNPs in 22 long non-coding RNAs (**Supplementary Table 1B**), and SNPs in 19 intergenic regions (**Supplementary Table 1C**). **Table 2** specifically highlights the exonic non-synonymous variations found in the various SNPs of the haploblock. Two variations found in the proteins NOTCH4 and DXO, respectively G534S and H261Q, are of particular interest due to their potential damaging effects, and they exhibit a strong LD with HLA-B*57:01 (**Table 2**). NOTCH4 is a transmembrane receptor of 2003 amino-acids, expressed ubiquitously, which has been associated with increased apoptosis in breast cancer (16) and increased inflammation in HIV-1 associated nephropathy (17). DXO is a decapping exoribonuclease, of size 396 AA, which leads to an increase of HIV-1 infectivity (18), its inactivation may thus favor protection against HIV-1 replication.

**Table 2:** List of non-synonymous variations linked to the haploblock.

SNP	Position	MAF CTR	p value	Gene	Protein mutation	PolyPhen-2 mutation report
rs7750641	31161533	11.4%	2.04E-08	*TCF19*	P109S	Benign (0%)
rs41558312	31411087	3.2%	2.08E-46	*MICA*	Q17R	Benign (9%)
rs41258944	32049465	5.9%	2.29E-14	*TNXB*	V3186I	Benign (11%)
rs41293883	31507043	3.6%	5.62E-38	*MICB*	T212I	Benign (6%)
rs12211410	32081646	9.2%	7.22E-13	*TNXB*	R1255H	Benign (0%)
rs2280801	31624287	5.5%	4.46E-19	*PRRC2A*	P106L	Benign (34%)
rs28732158	31662541	5.5%	4.48E-19	*GPANK1*	G266R	No prediction available
rs17207867	31970635	9.4%	1.27E-17	*DXO*	H261Q	Possibly damaging (66%)
rs8192591	32218019	3.3%	1.03E-17	*NOTCH4*	G534S	Possibly damaging (94%)

List of all the MHC SNPs in LD (r^2^>0.8) with SNPs of the HLA-B*57:01 haploblock in Europeans that corresponds to a non-synonymous variation of the encoded protein. The last column presents the protein impact of the amino-acid variation predicted by Polyphen-2 (in%), and one can remark damaging variations for the proteins NOTCH4 and DXO, the other variations seeming less impacting.

**Figure 3:**
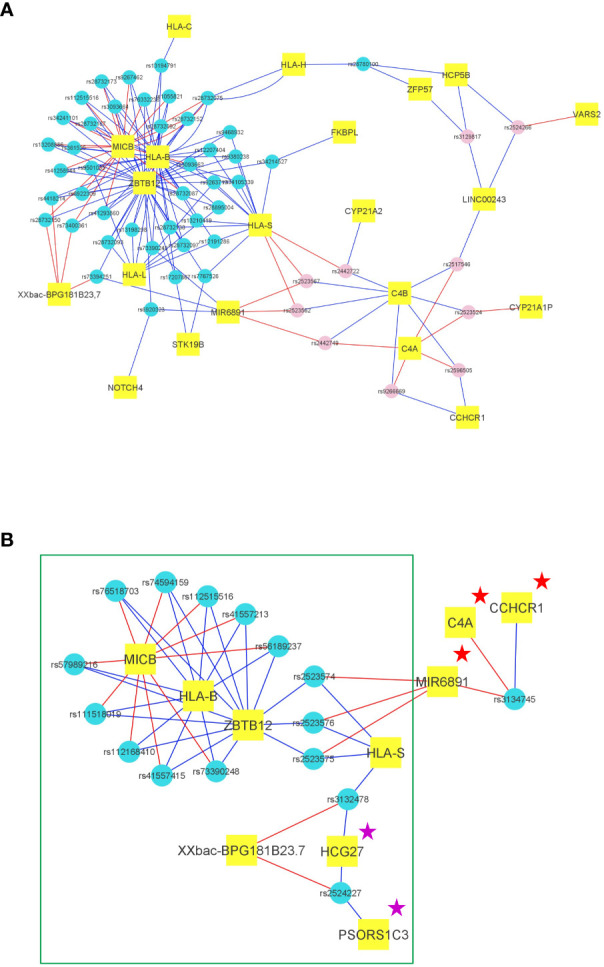
Transcriptional impact of the main SNPs of the haploblocks in the European and African American cohorts. **(A)** Representation of the transcriptional impact of the 54 representative SNPs of the HLA-B*57:01 haploblock in PBMCs according to GTEx. For each SNP (represented by small blue circles for the enriched alleles and pink circles for the decreased alleles), the 3 genes exhibiting the best p values for mRNA expression were selected, and each SNP was thus connected to 3 genes (represented in small rectangular tags). The link is blue when the SNP minor allele induces an increased mRNA expression, the link is red when the SNP minor allele induces a decreased mRNA expression. Hence, according to GTEx, 21 genes are differentially expressed by the 54 SNPs representing the HLA-B*57:01 haploblock. **(B)** Representation of the transcriptional impact of the 44 representative SNPs of the HLA-B*57:03 haploblock in African Americans in PBMCs according to GTEx. For each SNP (represented by small blue circles for the haploblock alleles), the 3 genes exhibiting the best p values for mRNA expression were selected, and each SNP was thus connected to 3 genes (represented in small rectangular tags). The link is blue when the SNP minor allele (or a SNP with r²>0,8) induces an increased mRNA expression, the link is red when the SNP minor allele (or a SNP with r²>0,8) induces a decreased mRNA expression. We observe a highly similar transcriptional profile for the African American HLA-B*57:03 SNPs and for the European HLA-B*57:01 SNPs marked by a green rectangle. Two proteins HCG27 and PSORS1C3 are marked by a magenta star because the mRNA are also expressed at a similar level in European HLA-B*57:01 individuals, but are not in the top 3. A Few discrepancies between Europeans and African Americans are observed for the mRNA expression of *C4A*, *Mir6891*, and *CCHCR1* which are marked by a red star.

We then decided to investigate the gene expression pattern associated with these SNP alleles. ECs, with their very low circulating virus, have a quasi-normal physiology. It is thus reasonable to use the GTex data (i.e. uninfected subjects) to deal with the level of mRNA expression in the PBMCs of ECs. **Supplementary Table 2A** presents the strongest differential expression patterns in PBMCs found for the 54 representative SNPs of the haploblock according to the GTEx database (19). As shown in **Supplementary Figure 1**, the ratio of the mean level of mRNA expression for the minor allele (which corresponds to the haploblock) over that of the major allele may reach extreme values under 0.5 or higher than 3, demonstrating the high impact of the haploblock on mRNA -and thus protein- expression. Twenty-one genes for which the differential mRNA expression linked to the SNP alleles of the haploblock yield strongly significant p values according to GTEx, are presented in **Figure 3A**. Among the overexpressed mRNAs, we observe *Mir6891* (p=10^-81^), *ZBTB12* (p=10^-64^), *NOTCH4* (p=10^-22^), *HLA-S* (p=10^-34^), *HLA-B* (p=10^-31^), *HLA-L* (p=10^-31^), and *C4A* (p=10^-22^). Conversely, *MICB* (p=10^-43^), *C4B* (p=10^-30^), and *ZFP57* (p=10^-26^) exhibit underexpression. The overexpression of HLA-B and underexpression of MICB, two well-known immune proteins associated with the HLA-B*57:01 haploblock, align well with expectations. The simultaneous increase of C4A and decrease of C4B is supposed to promote inflammatory cells (20). NOTCH4 is overexpressed in its mutated form associated with the haploblock minor alleles (**Table 2**), potentially reinforcing its inhibitory effect. ZBTB12 is a transcriptional factor of 459 residues, specifically expressed in PBMCs, with a potential role in hematopoietic development (21), known to regulate the human endogenous retroviruses (22). ZFP57 is a 452 AA protein involved in DNA methylation and imprinting during development, and a study has also shown its activity in adult PBMCs (23). Overall, we see that the haploblock alleles may induce a differential impact on numerous proteins with potential anti-viral or immune regulatory.

activities. This is a likely source of potent anti-HIV-1 response observed in carriers of HLA-B*57:01, in addition to its known role for antigen recognition and presentation.

### A similar haploblock associated with HLA-B*57:03 in African Americans

In African populations, the Minor Allele Frequency (MAF) of rs1131446 that tags HLA-B*57:03 is 0.037 in 1000Genomes, whereas the MAF of rs2395029 that tags HLA-B*57:01, is 0.004. Conversely, HLA-B*57:03 that has been associated with the control of viral load (24, 25) in Africans is nearly absent in Europeans. Since we had access to African American populations in ICGH, we tried to replicate the observations made in the European population. We analyzed 2 independent groups of HIV-1 positive subjects of African descent available from ICGH, CS1 and CS2, consisting of respectively 380 and 376 subjects (**Table 1A**). These groups were enriched with elite controllers (ECs) (**Table 1A**). In both CS1 and CS2, the SNP allele rs1131446-T on chromosome 6 showed the most significant association with viral load at setpoint by regression analysis (data not shown). This SNP exists in Europeans and is in strong LD with rs2395029 (R2 = 0.88, D’=0.96). In the two African American groups CS1 and CS2, rs1131446 had a MAF of 9%, whereas rs2395029 that tags HLA-B*57:01 was not observed. Rs1131446 represents a synonymous change at amino acid position 291 and serves as an excellent proxy for the HLA-B*57:03 allele, as LD assessment between rs1131446 and sequence-based HLA types in a subset of the ICGH sample of African descent (n=789) confirmed that the T allele is indeed linked to HLA-B*57:03 (r^2^ = 0.81).

**Figure 4:**
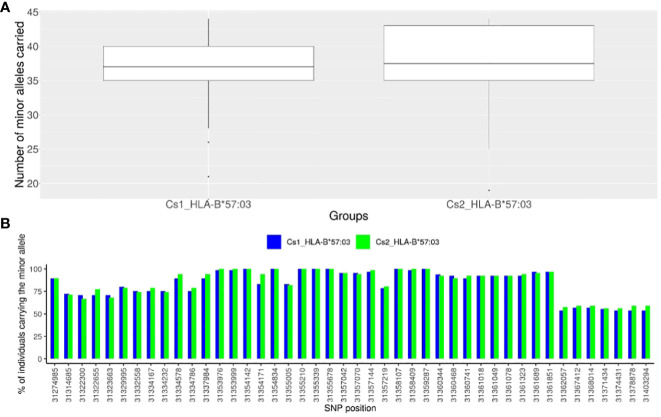
Description of the representative SNPs of the African haploblock. **(A)** Box-plot presenting the numbers of minor alleles of the 44 SNPs in high LD with rs1131446 (tagging HLA-B*57:03 in African Americans) carried for each individual in a given group: HLA-B*57:03 individuals of the Cs1 cohort in blue, and HLA-B*57:03 individuals of the Cs2 cohort in green. We see that most of the minor alleles of the 44 SNPs are carried simultaneously by the HLA-B*57:03 individuals of the Cs1 and Cs2 groups (median of the box-plot = 37 for the CS1 and CS2 cohorts, out of 44 SNPs). **(B)** For each of the 44 SNPs of the HLA-B*57:03 haploblock, histogram presenting the percentage of individuals carrying the minor allele among HLA-B*57:03 African American subjects: in blue for the Cs1 group, in red for the Cs2 group. One can see that most of the SNP minor alleles are carried by a majority of HLA-B*57:03 subjects, thus forming a haploblock. The SNPs are represented by their localization in chromosome 6 (GRCh38) on the x-axis.

To identify SNPs in LD with rs1131446 in both Cs1 and Cs2 populations, we considered all SNPs with an r^2^ value greater than 0.2 and a D’ value greater than 0.8. Through this analysis, we found a total of 44 SNPs that were located between positions 31,274,985 and 31,403,294 on chromosome 6, spanning a distance of 128 kilobases. As shown in **Figure 4A**, the African American individuals who carry the minor allele rs1131446-T, marking HLA-B*57:03, carry simultaneously most of the 44 SNP minor alleles which are in strong LD (median is 37 SNPs carried out of 44). Reciprocally, each one of the 44 SNP minor alleles are carried by most HLA-B*57:03 carriers in both Cs1 and Cs2 groups (**Figure 4B**).

**Figure 3B** presents the mRNA expression pattern in PBMCs for the 44 SNPs, as determined by the GTEx database (see methods). Importantly, a very similar pattern to the one observed for the SNPs of the HLA-B*57:01 haploblock (**Figure 3A**) was found, with the same differentially transcribed proteins and non-coding RNAs (**Figure 3B** and **Supplementary Table 2B**), including MICB, HLA-B, HLA-S, ZBTB12, PSORS1C3, HCG27, and XXbacBPG181B23.7. A discrepancy was nevertheless observed at the expression level of the 3 mRNA *C4A*, *CCHCR1*, and *Mir6891*. The analysis of the functional impact of all the genetic variants in LD with the SNPs of the haploblock revealed no damaging protein mutations. There were only 3 exonic variations observed that were localized in *HLA-B* and corresponded to the allele HLA-B*57:03, the other genetic variations annotated in the identified haploblock are described in **Supplementary Tables 1D, E**. The similarity of mRNA expression patterns between Europeans and African Americans suggests a potential additional mechanism for HIV control that supplements the *HLA*-B*57 alleles response by inducing an antiviral state that limits HIV replication.

## Discussion

A previous genetic association study made by the ICGH consortium had shown there was no significant SNP difference between HIV-1 infected individuals and uninfected controls, except at the level of the *CCR5* gene region (9). Here, we have conducted a comprehensive analysis focusing specifically on the elite controller (EC) phenotype that we compared with uninfected controls, and we found 2,626 positive signals (p<10-8) out of 35,552 SNPs analyzed in the *MHC* region. By looking for the single nucleotide polymorphisms (SNPs) differentially enriched between stratified ECs: the EC3 group (viral load between 100 and 1,000 copies/ml) and the EC2 group (viral load below 100 copies/ml), we could sub-select 379 SNPs specifically associated with the control of viral load. Unsurprisingly, the strongest signal found was the famous SNP rs2395029, known to tag HLA-B*57:01. There is indeed an enrichment of carriers of this allele among ECs which could be up to 6 times compared to a control population (**Table 1A**). Further investigation of SNPs in LD with rs2395029 revealed us the presence of a previously unknown large haploblock spanning 1.9 megabases (MB), strongly associated with HLA-B*57:01 in individuals of European descent. The existence of such a substantial haploblock is surprising given the high level of polymorphism in this region. This haploblock corresponds to the 57.1 ancestral haplotype previously described for classical *HLA* alleles (26). Our investigation has revealed that this ancestral haplotype involves in fact many more gene variants of the *MHC* region and likely contributes to the modification of biological processes involving numerous genes and long non-coding RNAs (lncRNAs), either through mutational effects (**Table 2**) or differential expression (**Figure 3** and **Supplementary Figure 1**).

Significantly, we extended our study to include two independent cohorts of African Americans, a population known to benefit from HLA-B*57:03-mediated protection. Intriguingly, we discovered that the primary SNP associated with viral load control, rs1131446, which tags HLA-B*57:03, was also linked to a haploblock comprising 44 SNPs in strong linkage disequilibrium, spanning a genomic distance of 128 kilobases. Analysis of mRNA expression patterns associated with this HLA-B*57:03 haploblock in African Americans, using data from GTEx, revealed a striking similarity to the mRNA expression pattern observed in the HLA-B*57:01 haploblock among Europeans (**Figures 3A, B**). The small discrepancy observed for the mRNA expression of *C4A*, *CCHCR1*, and *Mir6891* is of interest since it suggests that these proteins may not be directly involved in the effect of the HLA-B*57 haploblock on elite control.

The remarkable convergence of mRNA/protein-level impacts between these haploblocks in individuals of European and African descent suggests they could be a common mechanism contributing to the massive effect of HLA-B*57 haplotypes on HIV-1 control. Due to its anti-retroviral activity (ZBTB12, MICB, HLA-B), and to its conservation across ancestries (shared by Europeans and Africans), one could foresee that this haploblock was perhaps involved in the control of retroviruses knowing that the human genome and particularly the MHC region contains an important proportion of endogenous retrovirus (27).

We have thus confirmed the massive effect of HLA-B*57:01 for EC and found a large haploblock that provides new clues to explain this effect. One of our initial queries was why only 1-2% of the HLA-B*57:01 subjects ultimately become EC? To investigate this, we examined potential genetic variations between HLA-B*57:01 CTR and HLA-B*57:01 ECs, but we found no significant differences among the *HLA* common genetic variants between these two groups. The effect of HLA-B*57:01 for the control of HIV-1 replication at viral setpoint (meaning in average 18 months after infection by HIV-1) has been demonstrated with numerous cohorts of seropositive subjects, often involving no more than 500 seropositive subjects (6, 24). The small proportion of elite controllers in such cohorts (0.2% to 0.5% of EC would correspond approximately to 2 or 3 subjects) cannot solely account for the robust association observed in these cohorts for HLA-B*57:01 in relation to viral load control during the early stages of infection (viral setpoint). To explain this robust association, it is thus necessary to consider all carriers of the HLA-B*57 haploblock (a few dozen subjects, given a minor allele frequency of 3% in Europeans and 8% in African Americans). Therefore, it is likely that the haploblock has antiviral effects against HIV-1 in all individuals carrying the HLA-B*57:01 allele during the early stages of infection and it is not surprising to see a similar SNP distribution within the haploblock in both the control group and the elite controller group (**Figure 2**). This suggests a shift in our understanding, where this haploblock provides early protection to all carriers, followed by various disease progression patterns that may eventually lead to the elite controller phenotype. A majority of haploblock carriers will likely face additional factors, whether environmental or genetic, that will ultimately result in disease progression and prevent them from achieving EC status. Otherwise, all individuals carrying the HLA-B*57:01 allele would become ECs. For example, the initial infectious dose could be a critical factor, as a low infectious dose could facilitate the antiviral activity of the haploblock, or on the contrary a higher infectious dose could facilitate viral dissemination despite the antiviral activity conferred by the haploblock. Another impacting factor could be the presence of rare *HLA* genetic variants -which were not considered in this study- yielding for some individuals additional protection alongside the haploblock. Further research examining genetic differences between HLA-B*57:01 or HLA-B*57:03 CTR and HLA-B*57:01 or HLA-B*57:03 ECs across the entire genome may also help answer this latter question in the future.

In summary, our study has uncovered an haploblock and several protein layers that contribute to the elite controller phenotype of HLA-B*57 individuals. A key framework emerges, involving the interplay of direct antiviral factors such as the ZBTB12 protein, and the modulation of innate/adaptive immune responses through increased transcription of *HLA-B* and decreased transcription of *MICB*. The identification of this haploblock within the major histocompatibility complex (*MHC*) region raises broader questions for future research, such as why the haploblock in Europeans is larger than that in Africans. Additionally, it prompts exploration of haploblocks in Asian populations and the potential existence of other multifunctional haploblocks within the *MHC* region.

## Materials and methods

**Figure 5:**
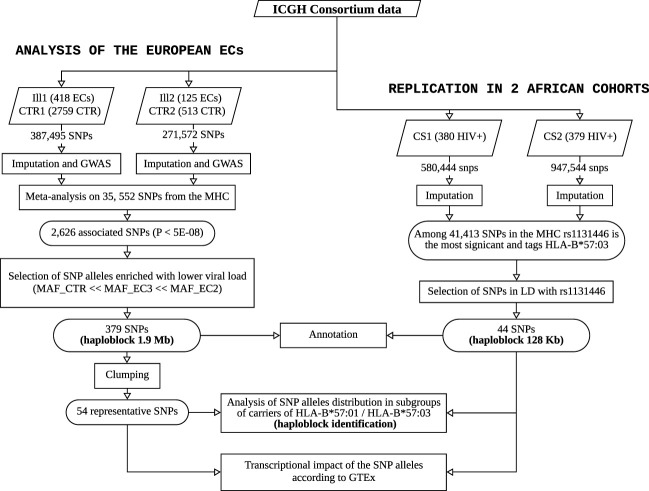
Flowchart summarizing the study and its main steps. The left panel shows the workflow performed on the ECs of European descent from ICGH to identify and characterize the haploblock linked to HLA-B*57:01, and the right panel describes the replication study performed to identify a similar haploblock linked to HLA-B*57:03 in two African American cohorts from ICGH.

For sake of clarity, a flowchart summarizing the study and its main steps is provided in **Figure 5**.

### Participant phenotypes and case/control matching

In this study, we used genotyped data from the International Collaboration on HIV-1 Genomics (ICGH). The ICGH project, initiated in 2012 with the support of the National Institutes of Health, aimed to consolidate genomic datasets from HIV-1 infected individuals worldwide. The consortium comprised 26 cohorts of seropositive subjects genotyped on diverse platforms, representing four continents (US, Europe, Australia, Africa). Genotypes for uninfected control individuals were obtained from three participating centers, the Illumina genotype control database and the Myocardial Infarction Genetics Consortium (MIGen) (9, 28). Each dataset underwent preliminary quality control procedures before centralizing all the data for combined analysis. However, to ensure consistency, additional quality control measures were implemented once the data were submitted, as described in the initial publication of ICGH (9).

All the individual cohorts contributing to the ICGH effort obtained ethical approval from their respective country institutions. The initial publication of ICGH in 2013 focused on susceptibility to HIV-1 infection (9) and included a comparison of 6,300 seropositive individuals with 7,200 uninfected controls of European descent. A subsequent publication in 2015 investigated the genetic association with viral load setpoint among the 6,300 seropositive subjects of European descent (9). In these studies, the cohorts were categorized into six groups of matched cases and controls based on genotyping platforms and geographic origin.

The present study focuses on two groups of Elite Controllers (EC) from the European cohorts, characterized by a viral load of less than 1,000 copies/mm3. Sufficient cases were available for our analysis in ill1 (418 ECs) and ill2 (125 ECs). Corresponding uninfected matched controls included 2,759 subjects in CTR1 and 513 subjects in CTR2. The matching process was described in the initial ICGH publication (9).

To replicate the findings related to the HLA-B*57 haploblock found in the European population, we included two African American cohorts of seropositive subjects available within the ICGH consortium: cs1, consisting of 380 seropositive individuals genotyped with the Illumina chip 650, and cs2, consisting of 379 seropositive individuals genotyped with the Illumina chip 1M. Both cs1 and cs2 cohorts encompass Elite Controllers (ECs) with viral load levels below 1,000 copies/ml copies/ml, with 110 and 89 ECs, respectively, as well as regular seropositive individuals.

A summary of the various groups utilized in this study is provided in **Table 1A**.

### Proportion of elite controllers and proportion of HLA-B*57 carriers among ECs

The GRIV cohort, which includes Long-Term Non-Progressors (LTNP) and Elite Controllers (ECs), is part of the ICGH consortium (9). The GRIV cohort was collected in France based on the LTNP phenotype, defined as documented infection for more than 8 years, maintaining a T lymphocyte count above 500/mm3, and no clinical symptoms (29). Among the 250 GRIV LTNPs with documented viral load, 50 (20%) were identified as ECs with a viral load below 1,000/mm3. At the time of data collection of the GRIV cohort, it was estimated that LTNP represented 1 to 2% of the known HIV-1 infected patients. Consequently, the ECs in the GRIV cohort correspond approximately to 0.2 to 0.4% of the known seropositive patients. This observation aligns well with the findings reported by Okulicz et al. in 2011 (2).

Knowing that 30% of the GRIV ECs carry the HLA-B*57:01 allele, while the prevalence of HLA-B*57:01 in the general French population is 6%, this suggests that the proportion of ECs among HLA-B*57:01 subjects falls within the range of 1 to 2%.

### Participant genotypes

The participants of European descent within the ICGH consortium were organized into matched case-control groups using a two-stage case/control matching strategy, as outlined in the initial publication of ICGH (9). This resulted in four clusters: Group 1 consisted of participants from the Netherlands genotyped on the Illumina platform, Group 2 included participants from France genotyped on the Illumina platform, Groups 3 and 4 encompassed participants from North America and non-Dutch/non-French European regions genotyped on the Illumina platform, and Groups 5 and 6 comprised participants from North America and non-Dutch/non-French European regions genotyped on the Affymetrix platform.

The ill1 EC group and its matching controls (CTR1) originated from Group 3, while the ill2 EC group and its matching controls (CTR2) were derived from Group 4. Genotypic data for these groups were generated using three genotyping arrays: Illumina 550, Illumina 650, and Illumina 1M. Prior to analysis, we conducted standard quality control (QC) procedures on each set (CTR1/ill1 and CTR2/ill2) to ensure the use of clean data.

Specifically, we filtered out monomorphic or rare variants with a minor allele frequency (MAF) less than 1%, as well as structural variations such as insertion-deletions. We also excluded sites with a missingness rate above 0.02 or a Hardy-Weinberg equilibrium (HWE) p-value lower than 10-6. Moreover, we examined the consistency between recorded sex information and sex inferred from the X chromosome, assessed heterozygosity and homozygosity rates, and verified the level of relatedness by calculating identity by descent (IBD). A similar QC process was separately conducted for the Cs1 and Cs2 groups of African American descent, who were genotyped on the Illumina 650 and 1M platforms.

### Imputation

Before imputation, additional quality control (QC) steps were applied to the two sets of European descent (ill1/CTR1 and ill2/CTR2). These QC measures utilized the checkbim steps of the McCarthy Group Tools (30), which involved removing single nucleotide polymorphisms (SNPs) with A/T and G/C alleles if the minor allele frequency (MAF) exceeded 40%. SNPs with discordant alleles, those with more than a 20% difference in allele frequency, and SNPs not present in the 1000 Genomes reference panel were also excluded. These QC steps ensured the utilization of high-quality data for the subsequent imputation process, enabling accurate results.

For ill1/CTR1, a total of 387,495 common SNPs were imputed, while for ill2/CTR2, 271,572 common SNPs were imputed. The imputation process followed a specific protocol: phasing was performed using Eagle2.4 (31), and imputation was carried out on the TOPMed Imputation Server (32) using Minimac4 (33) and the TOPMed reference panel (34).

The African American groups Cs1 and Cs2 underwent a similar imputation protocol, conducted separately from the European groups.

### Stratification

To assess patterns of population structure in the ill1/CTR1 and ill2/CTR2 datasets, a principal component analysis (PCA) was conducted. The analysis aimed to quantify the genetic ancestry of the participants. A set of 510,420 informative SNPs for ill1/CTR1 and 523,850 informative SNPs for ill2/CTR2 was selected for ancestral origin determination. To mitigate the influence of linkage disequilibrium, pruning was applied using an r^2^ threshold of 0.3, a sliding window size of 50, and a step size of 5. Regions on chromosomes 6, 8, and 17 with high linkage disequilibrium were excluded from the analysis.

The results of the PCA confirmed the homogeneity of both the ill1/CTR1 and ill2/CTR2 datasets, consistent with the findings reported in the first ICGH publication (9).

### Association testing by meta-analysis

For the ill1/CTR1 and ill2/CTR2 datasets, a logistic regression analysis comparing Elite Controllers (ECs) and controls (Ill1 vs CTR1 and ill2 vs CTR2) was conducted using SNPtest (35). The analysis focused on 35,552 variant dosages from the Major Histocompatibility Complex (MHC) region under an additive model. To account for population structure and minimize potential confounding effects, the first five principal components (PCs) were included as covariates in both analyses.

Following the individual dataset analyses, a meta-analysis was performed using GWAMA software (36) to combine the p-values from each dataset. The meta-analysis aimed to identify significant associations that satisfy the following conditions: a combined p-value less than or equal to 5.10^-8^, both individual p-values less than 0.05, an infotest value greater than 0.75 (indicating a good imputation quality), and the effect sizes (OR) in the same direction across datasets.

### Selection of SNPs significantly associated with EC and EC2

As mentioned in the previous paragraph, after conducting the meta-analysis on the set of 35,552 SNPs imputed in the *MHC* region, we identified 2,626 significant SNPs. In our analysis, we focused on SNPs with a minor allele frequency (MAF) that was enriched among subjects with the lowest viral loads. To quantify this enrichment, we calculated an enrichment score (ES) between the EC3 group (viral load between 100 and 1,000 copies/ml) and the EC2 group (viral load below 100 copies/ml).

Equation (1) defines the enrichment score ( $E_{score}$ ) as the percentage increase in the SNP’s minor allele frequency (MAF).


(1) $$MAF_{ctr}>MAF_{cv3}>MAF_{cv2}\;\text{or}\;MAF_{ctr}<MAF_{cv3}<MAF_{cv2}$$

$$E_{score}=\frac{MAF_{cv2}-MAF_{cv3}}{MAF_{cv3}}*100$$

MAFcv2 = MAF of the SNP in EC with viral load< 100 copies/ml.

MAFcv3 = MAF of the SNP in EC with 100 copies/ml< viral load< 1000 copies/ml.

MAFctr = MAF of the SNP in controls (uninfected).

Following the identification of 2,626 significant SNPs from the meta-analysis, we further examined the subset of SNPs that exhibited an increase (or decrease) in minor allele frequency (MAF) of more than 30% in EC2 compared to EC3 and controls. Out of the 2,626 significant SNPs, 379 SNPs met this criterion, indicating a substantial increase (or decrease) in MAF in EC2.

To work with a more manageable set of representative SNPs, we performed a clumping analysis using the program PLINK (37) on these 379 SNPs. The clumping analysis grouped together SNPs with an r^2^ correlation of 0.8 or higher and located within a physical distance of 200,000 base pairs. This clustering process resulted in a final set of 54 representative SNPs that could be used for further analysis.

Among these 54 representative SNPs, 44 exhibited a MAF increase greater than 30% between EC2 and EC3, corresponding to “protective” alleles. Additionally, 9 SNPs showed a MAF decrease greater than 30% between EC2 and EC3, corresponding to “risk” alleles. These representative SNPs provide a focused subset for subsequent investigations and analysis.

### Computation of the LD in various subgroups

To identify SNPs in linkage disequilibrium (LD) with SNP rs1131446, which serves as a marker for HLA-B*57:03 in African Americans, we employed the software PLINK (37) to conduct LD analysis on the genotypes of our cohorts. PLINK utilizes the maximum likelihood method to estimate LD coefficients, including the correlation coefficient (r²) and normalized LD coefficient (D’), between pairs of SNPs (38).

To establish appropriate threshold values for LD analysis, we referred to values observed in the European haploblock with SNP rs2395029, the reference marker for HLA-B*57:01 in Europeans. Based on this, we selected a threshold of an r² correlation coefficient greater than 0.2 and a D’ coefficient greater than 0.8 with rs1131446 in African Americans. We applied these criteria to both the Cs1 and Cs2 populations, and it resulted in the identification of 44 SNPs that exhibited significant LD with rs1131446 in both populations. These 44 SNPs provide valuable candidates for further investigation and analysis.

Consistent tools and methods were utilized for all r² calculations in our analysis, ensuring uniformity and comparability across the study.

### Score per individual and score per SNP allele, in each subgroup

For each individual (id), we computed 2 scores as the number of carried “protective” alleles, or alternatively, the number of carried “risk” alleles, from the 54 representative SNPs.

Knowing that 0 represents the alternate allele and 1, the reference allele, knowing that n is the number of protective or negative SNPs, knowing that P_s(0/0) and P_s(0/1) are the estimated posterior genotype probabilities for the SNP s imputed for individual id, we obtained a score per individual as presented in equation (2).


(2) $$Score_{id}=\sum_{s=1}^{s=n}P_{s}\left(0/0\right)+P_{s}\left(0/1\right)$$

We negate this score for under-enriched SNPs.

Each subgroup was represented by a boxplot in which each dot corresponded to the score of an individual (**Figure 2**). The Mann-Whitney U test was then employed to compare the distribution of the scores between subgroups.

For each SNP, it was also interesting to investigate the number of subjects carrying its minor allele in the various groups: the control group CTR, the elite control group EC (as well as EC2 and EC3), among the carriers of HLA-B*57:01 in Europeans or among the carriers of HLA-B*57:03 in African Americans (CTR_HLA-B*57:03, EC2_HLA-B*57:03, CS1_HLA-B*57:03, CS2_HLA-B*57:03). The HLA-B*57:01 carriers are those who have at least one G for SNP rs2395029, and the HLA-B*57:03 carriers are those who carry at least one T for SNP rs1131446 (since it is a good proxy of HLA-B*57:03 with r^2^>0,8).

For each SNP, we thus computed a score in a given subgroup, corresponding to the global percentage of subjects wearing its minor allele, as presented in equation (3).


(3) $$Score_{s}=\frac{\sum_{id=1}^{id=N}P_{s,id}\left(0/0\right)+P_{s,id}\left(0/1\right)}{N}*100$$

### Biological exploration of the SNPs with genetic annotation (Annovar)

We annotated the 379 SNPs from the European haploblock and the 44 SNPs from the African haploblock using Annovar (39) on dbSNP (avSNP150) (40) and the RefGene gene database (41). In order to include all mutants of interest, we considered in our analysis all SNPs with a r^2^ LD value greater than 0.8 with any SNP of the haploblock during the annotation process. We used Polyphen 2 (42) to predict the potential effect of amino acid substitutions on the structure and function of the protein for nonsynonymous SNPs identified.

### Transcriptional impact of the SNPs with GTEx

We used Ldexpress (43) from Ldlink (44), based on Genotype-Tissue Expression project data (GTEx, v8 release) (19), to investigate the genes significantly and differentially expressed in whole blood according to the alleles of the haploblock SNPs (as well as SNPs with r^2^ > 0.8). GTEx uses the genetic LD found in specified populations (either of European descent or of African descent) to select the SNPs of interest (with an r^2^>= 0.8 in our case), and then computes the differential transcription of genes for these SNPs among all samples (838 samples including 103 samples of African American descent).

For each SNP, we picked up the 3 genes most differentially expressed, i.e. that exhibited the lowest p-values.

The results obtained for the SNPs of the haploblock were then visualized in a network created with Cytoscape tool (45). On the representation of **Figure 3**, we use colours to present the direction of the enrichment in EC2 vs EC3 for the minor SNP allele (“protective” or “risk” allele), and the way by which it influences the mRNA gene expression in GTEx (increased or decreased expression).

## Data availability statement

The data presented in the study are deposited in the Figshare repository, 10.6084/m9.figshare.24615549.

## Ethics statement

Ethical approval for this study involving human genetic data was obtained for all cohorts by the local institutional review board of each group/center/study participating to the ICGH collective effort. All subjects provided written informed consent. The list of the ICGH cohorts/participants is as follows : 1. The AIDS clinical Trial Group (ACTG) in the USA 2. The AIDS Linked to the IntraVenous Experience (ALIVE) Cohort in Baltimore, USA 3. The Amsterdam Cohort Studies on HIV infection and AIDS (ACS) in the Netherlands 4. The ANRS CO18 in France 5. The ANRS PRIMO Cohort in France 6. The Center for HIV/AIDS Vaccine Immunology (CHAVI) in the USA 7. The Danish HIV Cohort Study in Denmark 8. The Genetic and Immunological Studies of European and African HIV-1+ Long Term Non-Progressors (GISHEAL) Study, in France and Italy 9. The GRIV Cohort in France 10. The Hemophilia Growth and Development Study (HGDS) in the USA 11. The Hospital Clinic-IDIBAPS Acute/ Recent HIV-1 Infection cohort in Barcelona, Spain 12. The Icona Foundation Study in Italy 13. The International HIV Controllers Study in Boston, USA 14. The IrsiCaixa Foundation Acute/Recent HIV-1 Infection cohort in Barcelona, Spain 15. The Modena Cohort in Modena, Italy 16. The Multicenter AIDS Cohort Study (MACS), in Baltimore, Chicago, Pittsburgh and Los Angeles, USA 17. The Multicenter Hemophilia Cohort Studies (MHCS) 18. The NCI Laboratory of Genomic Diversity in Frederick, USA 19. The Pumwani Sex Workers Cohort in Nairobi, Kenya, and Winnipeg, Canada 20. The San Francisco City Clinic Cohort (SFCCC) in San Francisco, USA 21. The Sanger RCC Study in Oxford, UK, and in Uganda 22. The Swiss HIV Cohort Study (SHCS), in Switzerland 23. The US military HIV Natural History Study (NHS) 24. The Wellcome Trust Case Control Consortium (WTCCC3) study of the genetics of host control of HIV-1 infection in the Gambia 25. The West Australian HIV cohort Study. The studies were conducted in accordance with the local legislation and institutional requirements.

## Author contributions

MR: Formal analysis, Investigation, Methodology, Software, Visualization, Writing – original draft, Data curation. LM: Data curation, Formal analysis, Visualization, Writing – original draft. J-LS: Formal analysis, Software, Validation, Writing – review & editing. MT: Data curation, Formal analysis, Visualization, Writing – review & editing. RM-S: Formal analysis, Methodology, Writing – review & editing. TL: Formal analysis, Data curation, Writing – review & editing. JN: Data curation, Writing – review & editing. RT: Formal analysis, Writing – review & editing. SL: Data curation, Validation, Writing – original draft. OD: Data curation, Writing – review & editing, Methodology. JF: Writing – review & editing, Funding acquisition, Project administration, Resources, Validation. AB: Writing – review & editing, Project administration, Supervision, Validation. SC: Data curation, Formal analysis, Writing – review & editing, Supervision. PM: Writing – original draft, Writing – review & editing, Data curation, Methodology, Resources, Validation, Visualization. J-FZ: Writing – original draft, Writing – review & editing, Investigation, Methodology, Validation, Visualization, Conceptualization, Funding acquisition, Project administration, Resources, Supervision.
