# Supplementary material for: The HLA-B*57:01 allele corresponds to a very large MHC haploblock likely explaining its massive effect for HIV-1 elite control
Source: Front Immunol. 2023 Dec 11;14:1305856. doi: 10.3389/fimmu.2023.1305856 (PMC10749428; doi:10.3389/fimmu.2023.1305856)
Supplement: Supplementary file 2 [file DataSheet_1.docx]

Supplementary Material

**Supplementary Table and figure legends**

**Supplementary Table 1: List of all the variations linked to the European and African American haploblocks**

1A. List of the genetic variations linked with the European haploblock SNPs (r^2^>0.8) located within a gene (less than 10 kb on the 5’ end, less than 2 kb in the 3’ end). 72 genes are concerned by such variants.

1B. List of the genetic variations linked with the European haploblock SNPs (r^2^>0.8) located within a Long non coding RNA. 22 long non coding RNA are concerned.

1C. List of the genetic variations linked with the European haploblock SNPs (r^2^>0.8) which are intergenic. They concern 19 genetic regions.

1D. List of the genetic variations linked with the African American haploblock SNPs (r^2^>0.8) located within a gene (less than 10 kb on the 5’ end, less than 2 kb in the 3’ end). 2 genes are concerned by such variants.

1E. List of the genetic variations linked with the African American haploblock SNPs (r^2^>0.8) which are intergenic. They concern 3 genetic region.

**Supplementary Table 2: Transcriptional impact of the SNPs linked to the European and African American haploblocks**

2A. List of the 54 SNPs of the European haploblock with their transcriptional impact in PBMCs according to GTEx (see Methods). The proteins presented for each SNP correspond to the ones with the 3 best GTEx p values. Of note, several p values reach 10^-30^, which is extremely significant.

2B. List of the 44 SNPs of the African-American haploblock with their transcriptional impact in PBMCs according to GTEx (see Methods). The proteins presented for each SNP correspond to the ones with the 3 best GTEx p values. Several p values reach 10^-20^.

**Supplementary Figure 1. Representation of the mRNA expression levels of some variants of the haploblock compared to their major allele**

This figure presents the normalized mRNA expression levels for a few gene variants and illustrates the impact of the HLA-B*57 haploblock variants on mRNA expression. The violin-plots of the mRNA expression levels of individuals according to their genotypes were obtained from the GTEx database (838 individuals) for the following genes : *ZBTB12, Mir6891, MICB, HLA-S, HLA-H, NOTCH4, C4A* and *C4B*. The number of individuals for each genotype is given, and the p value measures the statistical significance of the observed difference between the genotypes. In these plots, the mRNA expression levels are represented as a normalized base 2 logarithm, and one can compare the mRNA expression between the genotypes by simply subtracting the mean values and compute the power of 2 : for *ZBTB12* the ratio of mRNA expression between the genotypes was thus 3.3, for *Mir6891* 2.4, for *MICB* 0.39, for *HLA-S* 0.44, for *HLA-H* 2.11, for *NOTCH4* 2.24, for *C4A* 0.31, and for *C4B* 3.13.
