# Supplementary figures and images for: The HLA-B*57:01 allele corresponds to a very large MHC haploblock likely explaining its massive effect for HIV-1 elite control
Source: Front Immunol. 2023 Dec 11;14:1305856. doi: 10.3389/fimmu.2023.1305856 (PMC10749428; doi:10.3389/fimmu.2023.1305856)

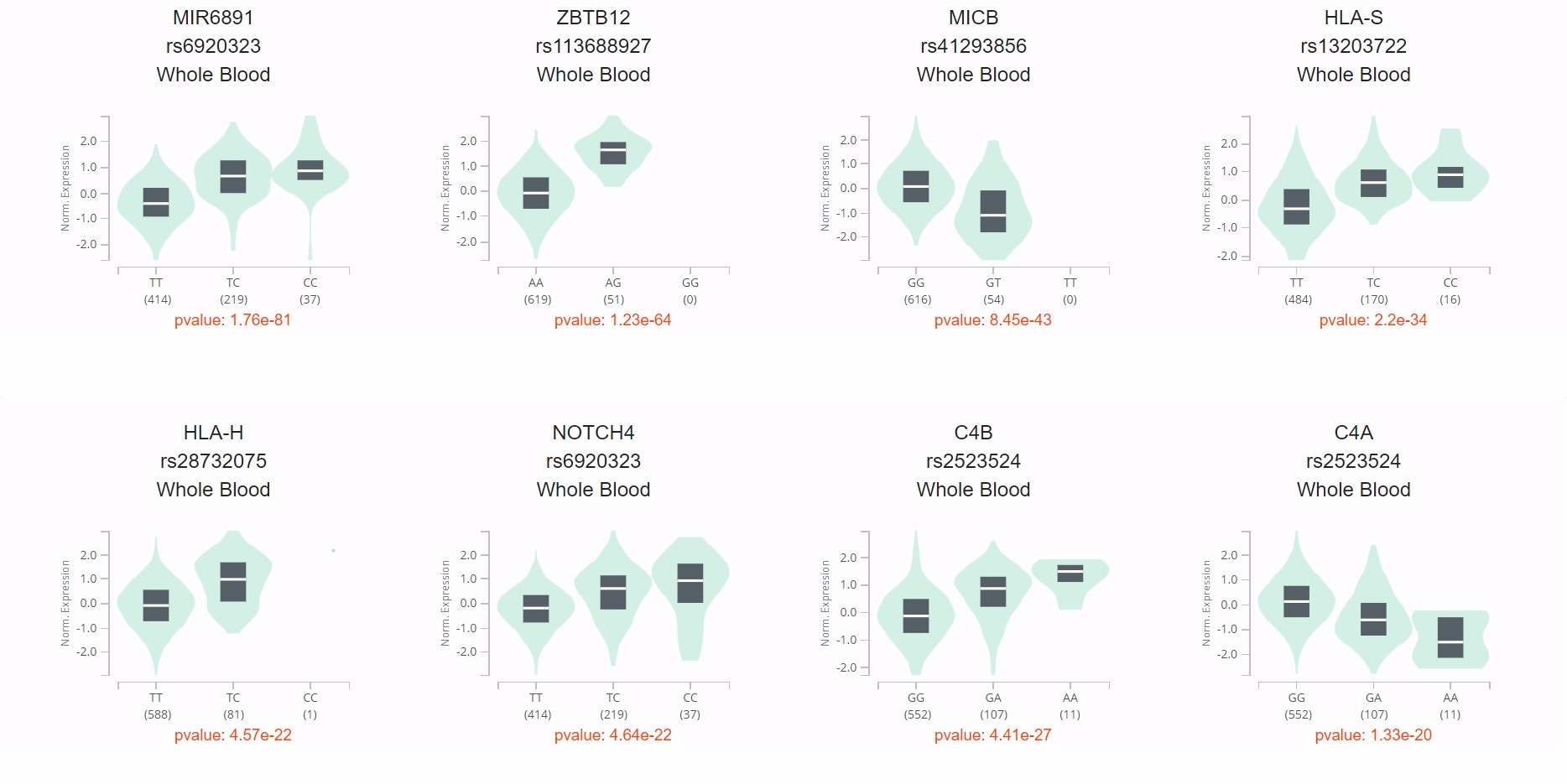

Supplement: Supplementary file 3 [file Image_1.jpeg]
